# Aluminum phosphide poisoning

**DOI:** 10.4103/0972-5229.58547

**Published:** 2009

**Authors:** Viroj Wiwanitkit

**Affiliations:** Wiwanitkit House, Bangkhae, Bangkok Thailand - 10160

Sir,

I read the recent publication by Jaiswal *et al*., on aluminium phosphide poisoning with great interest.[1] In their work,[1] correction of severe metabolic acidosis on patient outcome was studied. Jaiswal *et al*., noted that “there was significant improvement from 30.36% in the case when only half correction was done, as has been the common practice, to 57.5%, when full correction of metabolic acidosis was done.”[1] Indeed, aluminium phosphide poisoning is confirmed for induction of severe metabolic acidosis and relates to the high mortality. Khosla *et al*., noted that “severe metabolic acidosis was common and mortality was high (49%).”[2] The correction of metabolic acidosis seems to be the core therapeutic concept for aluminium phosphide poisoning. There is no doubt about the usefulness of this treatment. Several treatments are available at present, including gastric lavage with potassium permanganate solution, oral administration of charcoal and sorbitol suspension, intravenous administration of sodium bicarbonate, magnesium sulphate and calcium gluconate, and oral administration of sodium bicarbonate as well as alternative usage of oral administration of coconut oil.[3] No doubt that full correction of metabolic acidosis must be the aim of treatment since there is no reason to leave acidosis within the patient.
